# Interplay Between mTOR and Hippo Signaling in the Ovary: Clinical Choice Guidance Between Different Gonadotropin Preparations for Better IVF

**DOI:** 10.3389/fendo.2021.702446

**Published:** 2021-07-21

**Authors:** Kyriaki Papageorgiou, Eirini Mastora, Athanasios Zikopoulos, Maria E. Grigoriou, Ioannis Georgiou, Theologos M. Michaelidis

**Affiliations:** ^1^ Department of Biological Applications & Technologies, School of Health Sciences, University of Ioannina, Ioannina, Greece; ^2^ Institute of Molecular Biology and Biotechnology, Division of Biomedical Research, Foundation for Research and Technology – Hellas, Ioannina, Greece; ^3^ Laboratory of Medical Genetics of Human Reproduction, Medical School, University of Ioannina, Ioannina, Greece; ^4^ Medical Genetics and Assisted Reproduction Unit, Department of Obstetrics and Gynecology, Ioannina University Hospital, Ioannina, Greece; ^5^ Department of Molecular Biology & Genetics, Democritus University of Thrace, Alexandroupolis, Greece

**Keywords:** granulosa cells, r-hFSH, r-hLH, HP-hMG, ovarian stimulation, PI3K/mTOR/Akt, Hippo

## Abstract

One of the most widely used types of assisted reproduction technology is the *in vitro* fertilization (IVF), in which women undergo controlled ovarian stimulation through the administration of the appropriate hormones to produce as many mature follicles, as possible. The most common hormone combination is the co-administration of gonadotropin-releasing hormone (GnRH) analogues with recombinant or urinary-derived follicle-stimulating hormone (FSH). In the last few years, scientists have begun to explore the effect that different gonadotropin preparations have on granulosa cells’ maturation and apoptosis, aiming to identify new predictive markers of oocyte quality and successful fertilization. Two major pathways that control the ovarian development, as well as the oocyte–granulosa cell communication and the follicular growth, are the PI3K/Akt/mTOR and the Hippo signaling. The purpose of this article is to briefly review the current knowledge about the effects that the different gonadotropins, used for ovulation induction, may exert in the biology of granulosa cells, focusing on the importance of these two pathways, which are crucial for follicular maturation. We believe that a better understanding of the influence that the various ovarian stimulation protocols have on these critical molecular cascades will be invaluable in choosing the best approach for a given patient, thereby avoiding cancelled cycles, reducing frustration and potential treatment-related complications, and increasing the pregnancy rate. Moreover, individualizing the treatment plan will help clinicians to better coordinate assisted reproductive technology (ART) programs, discuss the specific options with the couples undergoing IVF, and alleviate stress, thus making the IVF experience easier.

## Introduction

The theory that FSH and luteinizing hormone (LH) are both required for the complete stimulation of follicular maturation and steroidogenesis was put forward 60 years ago from the Swedish scientist Bengt Falck (1). This idea was the basis for the stimulation of both hormonal systems for optimal follicular growth and maturation in IVF programs. Nowadays, ovarian stimulation during IVF includes the co-administration of GnRH analogues with the gonadotropins FSH, LH, and human chorionic gonadotropin (hCG).

A major drawback in IVF approaches is that the percentage of successful pregnancies is still low – approximately 27% pregnancies per IVF treatment in Europe (2, 3). Moreover, there is still a need for interventions to improve the initial recruitment and later survival of follicles to ensure good quality oocytes in healthy women, as well as in patients with poor ovarian response (POR), primary ovarian insufficiency (POI), or polycystic ovary syndrome (PCOS). Many studies compare the effects of different FSH-containing gonadotropin preparations in ovarian stimulation and IVF cycle outcomes, namely highly purified urinary human menopausal gonadotropin (HP-hMG) containing both FSH and LH activity, and recombinant human FSH (r-hFSH) alone or in combination with recombinant human LH (r-hLH). However, in most cases, the results are contradictory and inconclusive, and have led to controversial interpretations regarding the effectiveness of these gonadotropin regimens on follicular growth, antral follicle count, total oocytes retrieved, 2 pronuclear stage (2PN) oocytes, number of embryos, clinical pregnancy, and live birth rates in IVF (4–9). A pioneering study, a few years ago, demonstrated that the r-hFSH/r-hLH combination was more effective compared to HP-hMG, when the number of retrieved oocytes was high, also with regard to pregnancy rate per embryo transfer (10). Importantly however, a critical component of the stimulation regimens in IVF is the administration of a GnRH analogue, either agonist or antagonist to control the premature LH surge (11). Accordingly, an increasing number of studies reveal that the efficacy and the clinical outcomes of the different gonadotropin regimens appear to be dependent also, on the GnRH protocol used (9, 12–18). It is well known that the GnRH analogues can activate specific signal transduction pathways leading to distinct biological responses (19). Apparently, these treatments can alter the hormonal milieu, thereby favoring or hindering embryo quality and pregnancy rate (20). It is pertinent to note that FSH through binding to its cognate receptor FSHR (21), regulates the proliferation and differentiation of granulosa cells and prepares them to respond to gonadotropins and other endocrine signals, in order to undergo their final maturation. FSH is a glycoprotein, and it was recently shown that the hypo-glycosylated forms might be more efficient in promoting follicular growth and supporting granulosa cell survival *in vivo*, possibly by increasing serum estradiol levels (22). Interestingly, young women express partially glycosylated FSH whereas postmenopausal women express mainly the fully glycosylated form (23, 24), and this might influence both the biochemical properties and the efficacy of the various FSH preparations (25). This issue has been thoroughly discussed in a Delphi Consensus study recently (26).

## The Differential Effects of Gonadotropin Treatments on Oocyte – Granulosa Cell Communication and Follicular Maturation

Considering the vital role of granulosa cells in oocyte and follicle maturation, scientists have sought to investigate the influence of gonadotropin treatment on granulosa gene expression profiles. For example, the administration of r-hFSH, in comparison to HP-hMG (27) has been associated with higher expression of LH receptors and enzymes involved in the biosynthesis of steroids, and with lower mRNA levels of the FSH receptors in the granulosa cells (28). The presence of the FSH ligand (in cultured rat and bovine granulosa cells) leads to follicular activation and steroidogenesis, through the action of the highly conserved phosphoinositide-3 kinase (PI3K)/Akt/mammalian (or mechanistic) target of rapamycin (mTOR) and Hippo signaling pathways (29–31). The dysregulation of these pathways leads to increased apoptosis in ovarian cells (32, 33). Importantly, the incidence of apoptosis in granulosa cells has been linked to the quality of the oocytes and to the pregnancy outcome (34–36). There is some evidence indicating that the administration of HP-hMG increases the apoptosis of cumulus cells compared to r-hFSH or urinary FSH (37), and a recent study showed that high doses of r-hFSH suppress the apoptosis of granulosa cells in patients with endometriosis undergoing IVF (38). Therefore, researchers are currently exploring the consequences of the different protocols of gonadotropin ovarian stimulation on the apoptosis rate of granulosa cells (35, 39). However, in the ART clinical setting, more upstream effectors need to be considered since follicular growth is a dynamic and continuous process, characterized by a tightly regulated equilibrium between apoptosis and cell proliferation. For example, recently, it was elegantly shown that the FSH receptor synergizes with the G protein-coupled estrogen receptor (GPER), hence reprogramming FSH-induced death signals to proliferative stimuli that are important for nourishing oocyte survival (40). Heterodimerization of GPER with FSHR in granulosa cells switches the signaling mode from cAMP to pAKT activation, thereby positively affecting follicle maturation, and appears to correlate with the FSH responsiveness of patients undergoing IVF. This is particularly interesting, in light of evidence showing that estrogen can regulate Hippo signaling *via* GPER in breast tissue (41, 42). Accordingly, it might be more insightful to investigate the effects of the different gonadotropin preparations on the maturation of granulosa cells and the oocyte quality by monitoring the activity of the PI3K/Akt/mTOR and Hippo signaling cascades.

Although there are no studies yet comparing the effect of different gonadotropins on the Hippo pathway, there are data showing that r-hFSH and HP-hMG can differentially modulate the activities of the PI3K/Akt/mTOR signaling. For example, Ji et al., 2020 (43) using a GnRH antagonist protocol, observed that HP-hMG resulted in significantly higher insulin-like growth factor-1 (IGF-1) levels compared to r-hFSH on the day of oocyte retrieval, an effect that has been associated with better oocyte quality and pregnancy rate (44, 45). Interestingly, this was not the case in earlier studies when a GnRH agonist protocol had been employed (20, 46). The insulin/IGF-1 signaling pathway regulates the PI3K/mTOR/p70S6K cascade which as mentioned above plays an essential role in the FSH-mediated development of granulosa cells (30, 47, 48). This is important, also in light of recent findings showing that the hypo-glycosylated form of FSH, which is less abundant in the pituitary of postmenopausal women, activates more efficiently the PI3K/mTOR/p70S6K signaling (22).

Adding to the complexity of these interactions is the fact that there are many other signaling cues converging on both pathways. For example, other growth factors in addition to insulin/IGF-1, such as EGF, PDGF or VEGF are potent regulators of the PI3k/Akt/mTOR signaling in the follicles (49). Moreover, steroid hormones, like androgens which are the precursors for estrogen production, and known to stimulate granulosa and theca cell proliferation and to promote early antral follicle growth, can also regulate the expression of both FSH and IGF-1 receptor genes (50, 51). Furthermore, complex disorders such as the PCOS syndrome can affect the activation of both mTOR and Hippo signaling pathways. The development of PCOS has been associated with Hippo disruption and YAP overactivation leading to multiple early antral follicles and theca hyperplasia (49, 52). In addition, the expression of mTOR is elevated in a DHEA-treated PCOS animal model that could lead to insulin resistance, which is a characteristic of the PCOS phenotype (53). Other pathological conditions, such as endometriosis and ovarian cancer can exert an impact on the mTOR pathway by altering the expression of its targets  (54). Scientists have also noticed increased expression of YAP protein in mouse models with endometriosis whereas in mice treated with YAP inhibitors the endometriotic lesions were significantly decreased (55). Notably, the activation of mTOR pathway plays a fundamental role in the development of many autoimmune disorders (56), whereas Hippo signaling prevents autoimmunity and tissue damage (57, 58). In addition, vitamin D deficiency decreases mTOR activation in rat models (59) and human uterine fibroid cells (60). These are conditions that can influence the IVF outcomes (61–64). Future studies addressing the effects of the various gonadotropin combinations on the PI3k/Akt/mTOR and Hippo pathways in physiological conditions (including ageing) and disease states, are expected to increase our understanding of follicle development and develop personalized treatment plans that will help clinician’s decision and improve the success rate of IVF.

## The Interplay Between PI3K/Akt/mTOR Axis and Hippo Pathway in Follicular Development

The PI3K/Akt/mTOR axis is a key regulator of survival that fosters the processes of proliferation and differentiation, and inhibits apoptosis and autophagy (65, 66). The activation of this pathway is crucial for granulosa cell proliferation and follicular growth, especially during the primordial follicle development (67). Recent work from our lab revealed that the controlled pharmacological inhibition of the mTOR pathway in a rat experimental model can increase the number of competent primordial follicles while reducing atresia. Specifically, we showed that the follicles preserve their competence to resume growth two weeks after mTOR reactivation (68). Consistent with this, factors like Tsc1/2 and PTEN, which negatively regulate mTORC1, are capable to maintain the dormancy state of primordial follicles (69). Deregulation of these inhibitors leads to overactivation of the mTOR pathway that is linked to pathological situations where the entire pool of primordial follicles matures simultaneously resulting in an accelerated loss of primordial follicles and premature ovarian failure (POF) (70, 71). Over-activation of the mTOR pathway has been also associated with the emergence of PCOS and ovarian cancer (72). Importantly, however, there are no studies yet comparing the activation of mTOR pathway on granulosa cells obtained from IVF patients undergoing different protocols of gonadotropin stimulation.

Recent studies indicate that the Hippo signaling plays an instrumental role in the regulation of follicular growth. This pathway responds to mechanotransduction signals in order to maintain organ size through regulating cell proliferation and apoptosis (73, 74). The central components of the Hippo pathway are the kinases Mst1/2 and Lats1/2 which lead to the inactivation of its key downstream effectors Yes-associated protein (YAP) and transcriptional coactivator with PDZ-binding motif (TAZ) (75). When Hippo signaling is disrupted, YAP and TAZ translocate into the nucleus where they bind to the TEA Domain Transcription Factors (TEADs) promoting the expression of growth factors and apoptosis inhibitors (73, 76, 77). It has been reported that the development of primordial follicles is accompanied by an inhibition of the Hippo pathway (78, 79), while its overstimulation leads to a reduction in follicular proliferation and estrogen production in granulosa cells, both *in vivo*, and *in vitro* (80, 81). Before ovulation, oocyte-secreted factors contribute to the activation of YAP protein in granulosa cells stimulating their proliferation, whereas after ovulation, the Hippo pathway is transiently activated leading to YAP degradation, which allows the differentiation of granulosa cells into luteal cells and the production of progesterone (79).

There is an intrinsic mechanism that orchestrates the function of the mTOR and Hippo pathways through YAP and indirectly controls the granulosa cell–oocyte interactions. Interestingly, recent studies show that the communication of the Hippo pathway with the PI3K/Akt/mTOR axis and their coordinated regulation play a key role in follicular size and primordial maturity, through YAP and SMAD2/3 complex (48, 82, 83). Activation of the Akt/mTOR pathway using Akt stimulators in combination with inhibition of Hippo through ovarian fragmentation appears to increase the number of mature follicles in mouse models, but also in patients with POI or PCOS, adjusting follicular growth and ovulation, thereby leading to successful fertilization and pregnancy (49, 52, 84, 85). Cytoskeleton remodeling is one of the key factors regulating Hippo signaling and promoting the nuclear localization of YAP/TAZ complex (86). Importantly, recent findings in mouse models show that hMG administration leads to activation of the mTOR pathway (87), and GnRH induces cytoskeleton reorganization (a key process for the synthesis and secretion of gonadotropins) by activation of the mTOR kinase (88). Actin cytoskeleton dynamics mediates vital roles, also, for oocyte meiotic cell divisions through Hippo and mTOR signaling (89–91). In early stage oocytes (germinal vesicle) YAP is predominantly located in the cytoplasm, whereas during the subsequent stages of oocyte development (metaphase I), YAP becomes activated and translocates into the nucleus, suggesting a role of Hippo signaling in oocyte maturation (92). In addition, the mTOR pathway plays fundamental role on oocyte meiotic maturation through the activation of translation of specific mRNAs involved in spindle morphology and chromosomal alignment (93, 94). Consistently, disruption of mTOR signaling inhibits spindle migration and asymmetric division in mouse oocytes (95).

**Figure 1:**
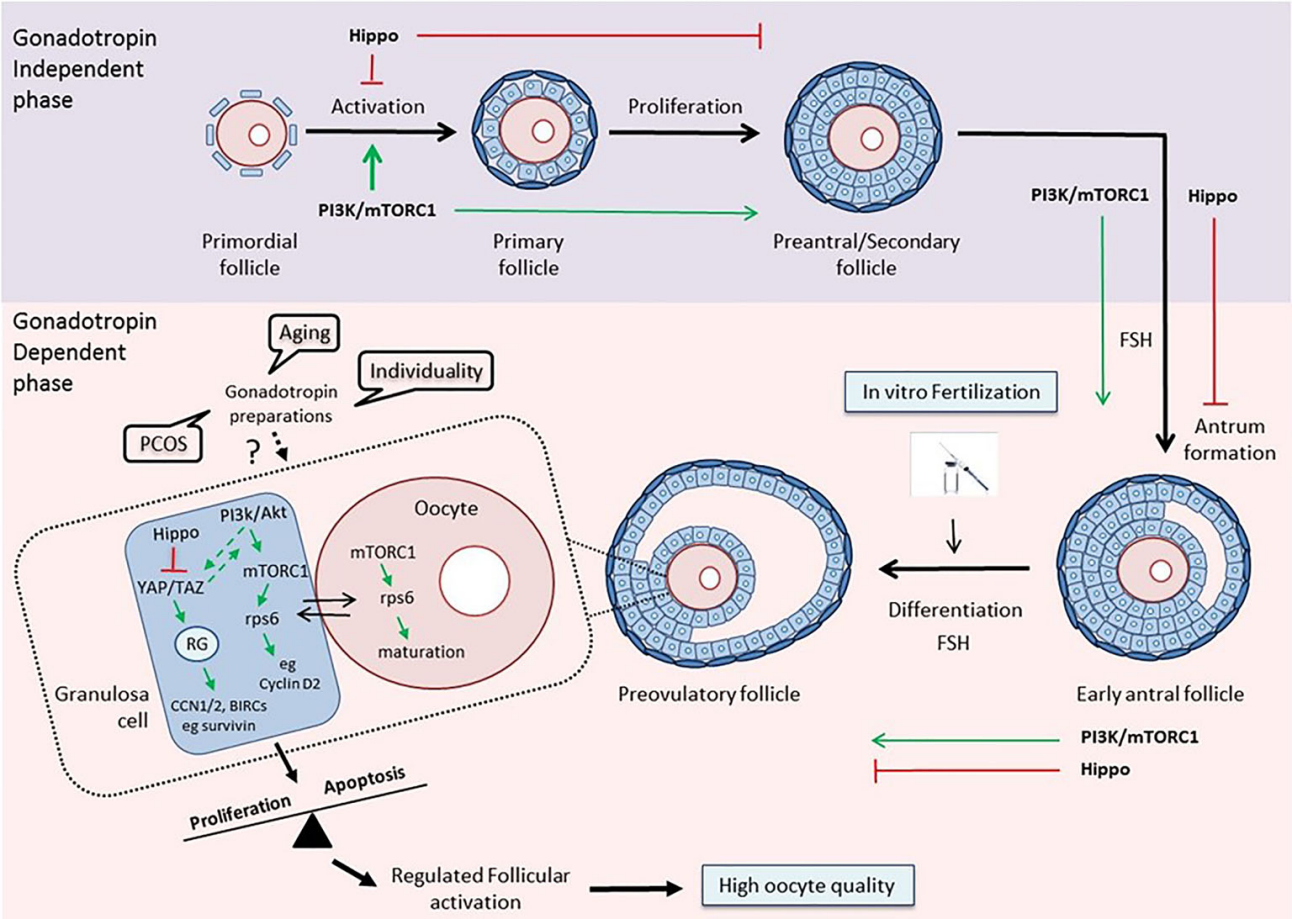
The PI3K/mTOR/Hippo pathways as guidance for clinical decision-making. Top: The PI3K/Akt/mTOR and Hippo pathways exert opposite effects on follicular development during the gonadotropin-independent phase. Activation of the PI3K pathway is crucial for each growing stage of the follicle, especially at the primordial and primary stages (30, 94). The Hippo pathway acts in a coordinated manner with PI3K in order to accelerate primordial follicle activation and promote follicular development (48). Bottom: The two pathways maintain their concerted action on follicular development during the gonadotropin-dependent phase of follicular growth, and especially on the maturation of granulosa cells and oocytes in the preovulatory follicles, thereby assuring regulated follicular activation and high oocyte quality (79, 96). Various disease states, aging, and the uniqueness of each woman, by influencing this balance, may affect the response to different gonadotropin preparations, and consequently, the outcome of the IVF. The activation status of key components of the PI3K and Hippo pathways may serve as a prognostic or predictive biomarker that can help clinicians guide treatment planning. (RG, Regulatory Genes).

Thus, it becomes evident from the above that a better understanding of the way that the different gonadotropin regimens affect the PI3K and Hippo pathways within the follicular environment in women with reduced ovarian reserve, polycystic ovary syndrome or advanced maternal age will allow their use as potential benchmarks for guidance of physicians regarding more efficient strategies for IVF (**Figure 1**).

## Concluding Remarks

It is clear, that further randomized controlled studies are needed to investigate the effects of the different gonadotropin preparations in the IVF outcome, and importantly, to combine both clinical and molecular attributes in order to appreciate the ovarian biological underpinnings of the various treatments. A better knowledge of the effects of the various gonadotropin preparations on the activation of follicles will allow the elaboration of appropriate biomarkers which in turn will render it possible to evaluate the efficacy of the different stimulation protocols in *in vitro* fertilization in different groups of patients. Current evidence reveals the presence of an active cross-talk between the PI3K/Akt/mTOR and the Hippo pathways, which is instrumentally involved in the regulated activation of primordial follicles, as well as, in follicular and oocyte growth. Consequently, a deeper understanding of the influence of the various ovarian stimulation protocols might exert on this interplay could help scientists to translate the emerging novel knowledge into clinical success and contribute to more efficient management of assisted reproduction methods. However, this is not an easy task. Despite the substantial progress in understanding ovarian follicular physiology, ART remains an inefficient process (97, 98). While the success rates of IVF/ART programs initially displayed an upward trend, the pregnancy and birth rates are declining in recent years (3). This issue has been thoroughly discussed by Norbert Gleicher and co-workers (99). Apparently, there are several causes, including potentially harmful add-ons to IVF practice, the woman’s age that dramatically influences the responses to exogenous gonadotropin stimulation (100–102) but also an evolving industrialization and commoditization of IVF (99). Considering the heterogeneity of the infertile population, understanding the best gonadotropin regimen for a particular patient necessitates two prerequisites. On the one hand, a personalized tailored approach (103, 104) which implies that we need to understand the mechanisms by which the same protocol results in different outcomes in different women, for example by monitoring gene expression profiles (105–108). On the other hand, the international cooperation between fertility societies such as ESHRE (European Society of Human Reproduction and Embryology), ASRM (American Society for Reproductive Medicine), or IFFS (International Federation of Fertility Societies) as well as Delphi Consensus statements, which by continuing to periodically update progress in basic research and reinforcing the dissemination of evidence-based information can facilitate and foster the translation of basic research into clinical practice.

In the long term, the elaboration of more straightforward and simple testing procedures based on key signaling cascades governing granulosa cell biology will help clinicians to prevent their patients from unnecessary treatment, and hopefully, will lead to more effective and individualized treatment protocols to improve birth rates.

## Author Contributions

KP and TM contributed to text conception. TM wrote the manuscript and KP has generated the figure. All authors contributed to the article and approved the submitted version.

## Conflict of Interest

The authors declare that the research was conducted in the absence of any commercial or financial relationships that could be construed as a potential conflict of interest.

## References

[1] Falck B. Site of Production of Estrogen in Rat Ovary as Studied in Micro-Transplants. Acta Phvsiol Stand (1959) 163:1–101. 10.1111/j.1748-1716.1960.tb01823.x 13821382

[2] Review of the 35th European Society of Human Reproduction and Embryology (ESHRE). Congress EMJ Repro Health (2019) 5(1):10–9.

[3] Kushnir VA Barad DH Albertini DF Darmon SK Gleicher N. Systematic Review of Worldwide Trends in Assisted Reproductive Technology 2004-2013. Reprod Biol Endocrinol (2017) 15(1):6. 10.1186/s12958-016-0225-2.3 28069012 PMC5223447

[4] Bleau N Agdi M Son WY Tan SL Dahan MH. A Comparison of Outcomes From *In Vitro* Fertilization Cycles Stimulated with Follicle Stimulating Hormone Plus Either Recombinant Luteinizing Hormone or Human Menopausal Gonadotropins in Subjects Treated with Long Gonadotropin Releasing Hormone Agonist Protocols. Int J Fertil Steril (2017) 1:79–84. 10.22074/ijfs.2017.4759 PMC5347454 28670424

[5] Levi-Setti PE Zerbetto I Baggiani A Zannoni E Sacchi L Smeraldi A. An Observational Retrospective Cohort Trial on 4,828 IVF Cycles Evaluating Different Low Prognosis Patients Following the POSEIDON Criteria. Front Endocrinol (2019) 10:282. 10.3389/fendo.2019.00282 PMC6517844 31139146

[6] Orvieto R. HMG Versus Recombinant FSH Plus Recombinant LH in Ovarian Stimulation for IVF: Does the Source of LH Preparation Matter? Reprod BioMed Online (2019) 6:1001–6. 10.1016/j.rbmo.2019.08.010 31672439

[7] Xia X Shi Y Geng L Liu D Hou Z Lin H. A Cohort Study of Both Human Menopausal Gonadotropin (HMG) and Recombinant Luteinizing Hormone Addition at Early Follicular Stage in *In Vitro* Fertilization Outcome: A STROBE-Compliant Study. Med (Baltimore) (2019) 98:e15512. 10.1097/MD.0000000000015512 PMC6531138 31083194

[8] Witz CA Daftary GS Doody KJ Park JK Seifu Y Yankov VI. Menopur in GnRH Antagonist Cycles With Single Embryo Transfer – High Responder (MEGASET-HR) Trial Group. Randomized, Assessor-Blinded Trial Comparing Highly Purified Human Menotropin and Recombinant Follicle-Stimulating Hormone in High Responders Undergoing Intracytoplasmic Sperm Injection. Fertil Steril (2020) 2:321–30. 10.1016/j.fertnstert.2020.03.029 32416978

[9] Tayyar AT Kahraman S. Comparison Between Cycles of the Same Patients When Using Recombinant Luteinizing Hormone + Recombinant Follicle Stimulating Hormone (rFSH), Human Menopausal Gonadotropin + rFSH and rFSH Only. Arch Med Sci (2019) 15(3):673–9. 10.5114/aoms.2017.72408 PMC6524176 31110533

[10] Revelli A Pettinau G Basso G Carosso A Ferrero A Dallan C. Controlled Ovarian Stimulation with Recombinant-FSH Plus Recombinant-LH vs. Human Menopausal Gonadotropin Based on the Number of Retrieved Oocytes: Results from a Routine Clinical Practice in a Real-Life Population. Reprod Biol Endocrinol (2015) 13:77. 10.1186/s12958-015-0080-6 26209525 PMC4514947

[11] Engel JB Schally AV. Drug Insight: Clinical Use of Agonists and Antagonists of Luteinizing-Hormone-Releasing Hormone. Nat Clin Pract Endocrinol Metab (2007) 3(2):157–67. 10.1038/ncpendmet0399 17237842

[12] Ye H Huang G Pei L Zeng P Luo X. Outcome of In Vitro Fertilization Following Stimulation with Highly Purified hMG or Recombinant FSH in Downregulated Women of Advanced Reproductive Age: A Prospective, Randomized and Controlled Trial. Gynecol Endocrinol (2012) 28(7):540–4. 10.3109/09513590.2011.650742 22390186

[13] Platteau P Nyboe Andersen A Loft A Smitz J Danglas P Devroey P. Highly Purified HMG Versus Recombinant FSH for Ovarian Stimulation in IVF Cycles. Reprod BioMed Online (2008) 17(2):190–8. 10.1016/s1472-6483(10)60194-0 18681992

[14] Kilani Z Dakkak A Ghunaim S Cognigni GE Tabarelli C Parmegiani L. A Prospective, Randomized, Controlled Trial Comparing Highly Purified hMG With Recombinant FSH in Women Undergoing ICSI: Ovarian Response and Clinical Outcomes. Hum Reprod (2003) 18(6):1194–9. 10.1093/humrep/deg252 12773445

[15] Coomarasamy A Afnan M Cheema D van der Veen F Bossuyt PM van Wely M. Urinary hMG Versus Recombinant FSH for Controlled Ovarian Hyperstimulation Following an Agonist Long Down-Regulation Protocol in IVF or ICSI Treatment: A Systematic Review and Meta-Analysis. Hum Reprod (2008) 23(2):310–5. 10.1093/humrep/dem305 18056719

[16] Shavit T Shalom-Paz E Samara N Aslih N Michaeli M Ellenbogen A. Comparison Between Stimulation With Highly Purified hMG or Recombinant FSH in Patients Undergoing IVF With GnRH Antagonist Protocol. Gynecol Endocrinol (2016) 32(8):629–33. 10.3109/09513590.2016.1153058 26939574

[17] Ana M Vicente M María RJ Trinidad GG Alberto R. Observational Study to Assess the Therapeutic Value of Four Ovarian Hyperstimulation Protocols in IVF After Pituitary Suppression With GnRH Antagonists in Normally Responding Women. Clin Med Insights Reprod Health (2011) 5:1–9. 10.4137/CMRH.S6339 24453506 PMC3888073

[18] Bosch E Vidal C Labarta E Simon C Remohi J Pellicer A. Highly Purified hMG Versus Recombinant FSH in Ovarian Hyperstimulation With GnRH Antagonists–a Randomized Study. Hum Reprod (2008) 23(10):2346–51. 10.1093/humrep/den220 18583332

[19] Millar RP Pawson AJ Morgan K Rissman EF Lu ZL. Diversity of Actions of GnRHs Mediated by Ligand-Induced Selective Signaling. Front Neuroendocrinol (2008) 29(1):17–35. 10.1016/j.yfrne.2007.06.002 17976709 PMC2667102

[20] Smitz J Andersen AN Devroey P Arce JC MERIT Group. Endocrine Profile in Serum and Follicular Fluid Differs After Ovarian Stimulation With HP-hMG or Recombinant FSH in IVF Patients. Hum Reprod (2007) 22(3):676–87. 10.1093/humrep/del445 17110397

[21] Ulloa-Aguirre A Zariñán T Jardón-Valadez E Gutiérrez-Sagal R Dias JA. Structure-Function Relationships of the Follicle-Stimulating Hormone Receptor. Front Endocrinol (Lausanne) (2018) 9:707. 10.3389/fendo.2018.00707 30555414 PMC6281744

[22] Hua G George JW Clark KL Jonas KC Johnson GP Southekal S. Hypo-Glycosylated hFSH Drives Ovarian Follicular Development More Efficiently Than Fully-Glycosylated hFSH: Enhanced Transcription and PI3K and MAPK Signaling. Hum Reprod (2021) 36(7):1891–906. 10.1093/humrep/deab135 PMC8213452 34059912

[23] Bousfield GR Butnev VY Rueda-Santos MA Brown A Hall AS Harvey DJ. Macro- and Micro-Heterogeneity in Pituitary and Urinary Follicle-Stimulating Hormone Glycosylation. J Glycomics Lipidomics (2014) 4:1000125. 10.4172/2153-0637.1000125 25722940 PMC4338580

[24] Butnev VY Butnev VY May JV Shuai B Tran P White WK. Production, Purification, and Characterization of Recombinant hFSH Glycoforms for Functional Studies. Mol Cell Endocrinol (2015) 405:42–51. 10.1016/j.mce.2015.01.026 25661536 PMC4378652

[25] Dias JA Ulloa-Aguirre A. New Human Follitropin Preparations: How Glycan Structural Differences May Affect Biochemical and Biological Function and Clinical Effect. Front Endocrinol (Lausanne) (2021) 12:636038. 10.3389/fendo.2021.636038 33815292 PMC8018285

[26] Orvieto R Venetis CA Fatemi HM D’Hooghe T Fischer R Koloda Y. Optimising Follicular Development, Pituitary Suppression, Triggering and Luteal Phase Support During Assisted Reproductive Technology: A Delphi Consensus. Front Endocrinol (Lausanne) (2021) 12:675670. 10.3389/fendo.2021.675670 34040586 PMC8142593

[27] Grøndahl ML Borup R Lee YB Myrhøj V Meinertz H Sørensen S. Differences in Gene Expression of Granulosa Cells From Women Undergoing Controlled Ovarian Hyperstimulation With Either Recombinant Follicle-Stimulating Hormone or Highly Purified Human Menopausal Gonadotropin. Fertil Steril (2009) 5:1820–30. 10.1016/j.fertnstert.2008.02.137 18439596

[28] Yi-Ming Z Shyamal KR. Downregulation of Follicle-Stimulating Hormone (FSH)-Receptor Messenger RNA Levels in the Hamster Ovary: Effect of the Endogenous and Exogenous FSH. Biol Reprod (2004) 6:1580–8. 10.1095/biolreprod.103.026898 14749302

[29] Kayampilly PP Menon KM. Follicle-Stimulating Hormone Increases Tuberin Phosphorylation and Mammalian Target of Rapamycin Signaling Through an Extracellular Signal-Regulated Kinase-Dependent Pathway in Rat Granulosa Cells. Endocrinology (2007) 8:3950–7. 10.1210/en.2007-0202 17510244

[30] Guo Z Yu Q. Role of mTOR Signaling in Female Reproduction. Front Endocrinol (Lausanne) (2019) 10:692. 10.3389/fendo.2019.00692 31649622 PMC6794368

[31] Plewes MR Hou X Zhang P Liang A Hua G Wood JR. Yes-Associated Protein 1 is Required for Proliferation and Function of Bovine Granulosa Cells In Vitro. Biol Reprod (2019) 5:1001–17. 10.1093/biolre/ioz139 PMC6877782 31350850

[32] Sun T Pepling ME Diaz FJ. Lats1 Deletion Causes Increased Germ Cell Apoptosis and Follicular Cysts in Mouse Ovaries. Biol Reprod (2015) 1:22. 10.1095/biolreprod.114.118604 26040669

[33] Hu X Xia M Wang J Yu H Chai J Zhang Z. Dual PI3K/mTOR Inhibitor PKI-402 Suppresses the Growth of Ovarian Cancer Cells by Degradation of Mcl-1 Through Autophagy. BioMed Pharmacother (2020) 129:110397. 10.1016/j.biopha.2020.110397 32585451

[34] Nakahara K Saito H Saito T Ito M Ohta N Takahashi T. The Incidence of Apoptotic Bodies in Membrana Granulosa Can Predict Prognosis of Ova From Patients Participating in *In Vitro* Fertilization Programs. Fertil Steril (1997) 68:312–7. 10.1016/s0015-0282(97)81521-x 9240262

[35] Regan SLP Knight PG Yovich JL Leung Y Arfuso F Dharmarajan A. Granulosa Cell Apoptosis in the Ovarian Follicle-A Changing View. Front Endocrinol (Lausanne) (2018) 9:61. 10.3389/fendo.2018.00061 29551992 PMC5840209

[36] Regan SLP Knight PG Yovich JL Stanger JD Leung Y Arfuso F. The Effect of Ovarian Reserve and Receptor Signalling on Granulosa Cell Apoptosis During Human Follicle Development. Mol Cell Endocrinol (2017) 470:219–27. 10.1016/j.mce.2017.11.002 29113831

[37] Requena A Cruz M Agudo D Pacheco A García-Velasco JA. Type of Gonadotropin During Controlled Ovarian Stimulation Affects the Endocrine Profile in Follicular Fluid and Apoptosis Rate in Cumulus Cells. Eur J Obstet Gynecol Reprod Biol (2016) 197:142–6. 10.1016/j.ejogrb.2015.12.018 26751823

[38] Wiweko B Yanuar Mohammad Y Muna N Mutia K Witjaksono J Purwito Adi N. A High Dose of Total Recombinant FSH Suppresses Granulosa Cell Apoptosis and Maintains Oocyte Quality in Endometriosis: A Cross-Sectional Study. F1000Research (2019) 8:93. 10.12688/f1000research.17058.1

[39] Almeida CP Ferreira MCF Silveira CO Campos JR Borges IT Baeta PG. Clinical Correlation of Apoptosis in Human Granulosa Cells-A Review. Cell Biol Int (2018) 10:1276–81. 10.1002/cbin.11036 30080285

[40] Casarini L Lazzaretti C Paradiso E Limoncella S Riccetti L Sperduti S. Membrane Estrogen Receptor (GPER) and Follicle-Stimulating Hormone Receptor (FSHR) Heteromeric Complexes Promote Human Ovarian Follicle Survival. iScience (2020) 23:101812. 10.1016/j.isci.2020.101812 33299978 PMC7702187

[41] Zhou X Wang S Wang Z Feng X Liu P Lv XB. Estrogen Regulates Hippo Signaling *via* GPER in Breast Cancer. J Clin Invest (2015) 5:2123–35. 10.1172/JCI79573 PMC4463207 25893606

[42] Girgert R Emons G Gründker C. Estrogen Signaling in ERα-Negative Breast Cancer: ERβ and GPER. Front Endocrinol (Lausanne) (2019) 9:781. 10.3389/fendo.2018.00781 30687231 PMC6333678

[43] Ji Z Quan X Lan Y Zhao M Tian X Yang X. Gonadotropin Versus Follicle-Stimulating Hormone for Ovarian Response in Patients Undergoing in vitro Fertilization: A Retrospective Cohort Comparison. Curr Ther Res Clin Exp (2019) 92:100572. 10.1016/j.curtheres.2019.100572 31908689 PMC6940711

[44] Fried G Remaeus K Harlin J Krog E Csemiczky G Aanesen A. Inhibin B Predicts Oocyte Number and the Ratio IGF-I/IGFBP-1 may Indicate Oocyte Quality During Ovarian Hyperstimulation for in vitro fertilization. J Assist Reprod Genet (2003) 20(5):167–76. 10.1023/a:1023656225053 PMC3455298 12812459

[45] Mehta BN Chimote NM Chimote MN Chimote NN Nath NM. Follicular Fluid Insulin Like Growth Factor-1 (FF IGF-1) is a Biochemical Marker of Embryo Quality and Implantation Rates in in vitro fertilization cycles. J Hum Reprod Sci (2013) 6(2):140–6. 10.4103/0974-1208.117171 PMC3778604 24082656

[46] Bilge M Ozdemirci S Esinler D Karahanoglu E Esinler I Aksu T. Assessment of Follicular and Serum VEGF and IGF-1 in ICSI Patients: hMG vs rFSH. Clin Exp Obstet Gynecol (2015) 42(5):576–9. 10.12891/ceog1896.2015 26524801

[47] Gloaguen P Crépieux P Heitzler D Poupon A Reiter E. Mapping the Follicle-Stimulating Hormone-Induced Signaling Networks. Front Endocrinol (Lausanne) (2011) 2:45. 10.3389/fendo.2011.00045 22666216 PMC3364461

[48] Grosbois J Demeestere I. Dynamics of PI3K and Hippo Signaling Pathways During *In Vitro* Human Follicle Activation. Hum Reprod (2018) 9:1705–14. 10.1093/humrep/dey250 30032281

[49] Hsueh AJ Kawamura K Cheng Y Fauser BC. Intraovarian Control of Early Folliculogenesis. Endocr Rev (2015) 1:1–24. 10.1210/er.2014-1020 PMC4309737 25202833

[50] Gleicher N Weghofer A Barad DH. The Role of Androgens in Follicle Maturation and Ovulation Induction: Friend or Foe of Infertility Treatment? Reprod Biol Endocrinol (2011) 9:116. 10.1186/1477-7827-9-116 21849061 PMC3170254

[51] Lebbe M Woodruff TK. Involvement of Androgens in Ovarian Health and Disease. Mol Hum Reprod (2013) 19(12):828–37. 10.1093/molehr/gat065 PMC3843026 24026057

[52] Maas K Mirabal S Penzias A Sweetnam PM Eggan KC Sakkas D. Hippo Signaling in the Ovary and Polycystic Ovarian Syndrome. J Assist Reprod Genet (2018) 10:1763–71. 10.1007/s10815-018-1235-0 PMC6150890 30120633

[53] Liu J Wu DC Qu LH Liao HQ Li MX. The Role of mTOR in Ovarian Neoplasms, Polycystic Ovary Syndrome, and Ovarian Aging. Clin Anat (2018) 31(6):891–8. 10.1002/ca.23211 29752839

[54] Rogers-Broadway KR Kumar J Sisu C Wander G Mazey E Jeyaneethi J. Differential Expression of mTOR Components in Endometriosis and Ovarian Cancer: Effects of Rapalogues and Dual Kinase Inhibitors on mTORC1 and mTORC2 Stoichiometry. Int J Mol Med (2019) 43(1):47–56. 10.3892/ijmm.2018.3967 30387804 PMC6257843

[55] Song Y Fu J Zhou M Xiao L Feng X Chen H. Activated Hippo/Yes-Associated Protein Pathway Promotes Cell Proliferation and Anti-Apoptosis in Endometrial Stromal Cells of Endometriosis. J Clin Endocrinol Metab (2016) 101(4):1552–61. 10.1210/jc.2016-1120 PMC4880175 26977530

[56] Perl A. mTOR Activation Is a Biomarker and a Central Pathway to Autoimmune Disorders, Cancer, Obesity, and Aging. Ann N Y Acad Sci (2015) 1346(1):33–44. 10.1111/nyas.12756 25907074 PMC4480196

[57] Du X Shi H Li J Dong Y Liang J Ye J. Mst1/Mst2 Regulate Development and Function of Regulatory T Cells Through Modulation of Foxo1/Foxo3 Stability in Autoimmune Disease. J Immunol (2014) 192(4):1525–35. 10.4049/jimmunol.1301060 24453252

[58] Li J Du X Shi H Deng K Chi H Tao W. Mammalian Sterile 20-Like Kinase 1 (Mst1) Enhances the Stability of Forkhead Box P3 (Foxp3) and the Function of Regulatory T Cells by Modulating Foxp3 Acetylation. J Biol Chem (2015) 290(52):30762–70. 10.1074/jbc.M115.668442 PMC4692206 26538561

[59] Lajtai K Nagy CT Tarszabó R Benkő R Hadjadj L Sziva RE. Effects of Vitamin D Deficiency on Proliferation and Autophagy of Ovarian and Liver Tissues in a Rat Model of Polycystic Ovary Syndrome. Biomolecules (2019) 9(9):471. 10.3390/biom9090471 PMC6770417 31509973

[60] Al-Hendy A Diamond MP Boyer TG Halder SK. Vitamin D3 Inhibits Wnt/β-Catenin and mTOR Signaling Pathways in Human Uterine Fibroid Cells. J Clin Endocrinol Metab (2016) 101(4):1542–51. 10.1210/jc.2015-3555 PMC4880168 26820714

[61] Dabrowski FA Grzechocinska B Wielgos M. The Role of Vitamin D in Reproductive Health–A Trojan Horse or the Golden Fleece? Nutrients (2015) 7(6):4139–53. 10.3390/nu7064139 PMC4488777 26035242

[62] Li S Hu L Zhang C. Urinary Vitamin D-Binding Protein as a Marker of Ovarian Reserve. Reprod Biol Endocrinol (2021) 19(1):80. 10.1186/s12958-021-00762-9 34074317 PMC8168315

[63] Chu C Tsuprykov O Chen X Elitok S Krämer BK Hocher B. Relationship Between Vitamin D and Hormones Important for Human Fertility in Reproductive-Aged Women. Front Endocrinol (Lausanne) (2021) 12:666687. 10.3389/fendo.2021.666687 33935976 PMC8081388

[64] Szeliga A Calik-Ksepka A Maciejewska-Jeske M Grymowicz M Smolarczyk K Kostrzak A. Autoimmune Diseases in Patients With Premature Ovarian Insufficiency-Our Current State of Knowledge. Int J Mol Sci (2021) 22(5):2594. 10.3390/ijms22052594 33807517 PMC7961833

[65] Hung CM Garcia-Haro L Sparks CA Guertin DA. mTOR-Dependent Cell Survival Mechanisms. Cold Spring Harb Perspect Biol (2012) 12:a008771. 10.1101/cshperspect.a008771 PMC3504431 23124837

[66] Laplante M Sabatini DM. mTOR Signaling at a Glance. J Cell Sci (2009) 122:3589–94. 10.1242/jcs.051011 PMC2758797 19812304

[67] Yu J Yaba A Kasiman C Thomson T Johnson J. mTOR Controls Ovarian Follicle Growth by Regulating Granulosa Cell Proliferation. PloS One (2011) 7:e21415. 10.1371/journal.pone.0021415 PMC3130037 21750711

[68] Pargianas M Kosmas I Papageorgiou K Kitsou C Papoudou-Bai A Batistatou A. Follicle Inhibition at the Primordial Stage Without Increasing Apoptosis, With a Combination of Everolimus, Verapamil. Mol Biol Rep (2020) 11:8711–26. 10.1007/s11033-020-05917-2 33079326

[69] Adhikari D Zheng W Shen Y Gorre N Hämäläinen T Cooney AJ. Tsc/mTORC1 Signaling in Oocytes Governs the Quiescence and Activation of Primordial Follicles. Hum Mol Genet (2010) 3:397–410. 10.1093/hmg/ddp483 PMC2798719 19843540

[70] Adhikari D Flohr G Gorre N Shen Y Yang H Lundin E. Disruption of Tsc2 in Oocytes Leads to Overactivation of the Entire Pool of Primordial Follicles. Mol Hum Reprod (2009) 12:765–70. 10.1093/molehr/gap092 19843635

[71] Adhikari D Risal S Liu K Shen Y. Pharmacological Inhibition of mTORC1 Prevents Over-Activation of the Primordial Follicle Pool in Response to Elevated PI3K Signaling. PloS One (2013) 1:e53810. 10.1371/journal.pone.0053810 PMC3543305 23326514

[72] Liu AL Liao HQ Li ZHL Liu J Zhou CL Guo ZF. New Insights Into mTOR Signal Pathways in Ovarian-Related Diseases: Polycystic Ovary Syndrome and Ovarian Cancer. Asian Pac J Cancer Prev (2016) 12:5087–94. 10.22034/APJCP.2016.17.12.5087 PMC5454641 28122439

[73] Pan D. Hippo Signaling in Organ Size Control. Genes Dev (2007) 21(8):886–97. 10.1101/gad.1536007 17437995

[74] Hergovich A. Mammalian Hippo Signalling: A Kinase Network Regulated by Protein-Protein Interactions. Biochem Soc Trans (2012) 1:124–8. 10.1042/BST20110619 PMC3398126 22260677

[75] Zheng Y Pan D. The Hippo Signaling Pathway in Development and Disease. Dev Cell (2019) 3:264–82. 10.1016/j.devcel.2019.06.003 PMC6748048 31386861

[76] Pocaterra A Romani P Dupont S. YAP/TAZ Functions and Their Regulation at a Glance. J Cell Sci (2020) 2:jcs230425. 10.1242/jcs.230425 31996398

[77] Reggiani F Gobbi G Ciarrocchi A Sancisi V. YAP and TAZ Are Not Identical Twins. Trends Biochem Sci (2021) 2:154–68. 10.1016/j.tibs.2020.08.012 32981815

[78] Xiang C Li J Hu L Huang J Luo T Zhong Z. Hippo Signaling Pathway Reveals a Spatio-Temporal Correlation With the Size of Primordial Follicle Pool in Mice. Cell Physiol Biochem (2015) 3:957–68. 10.1159/000369752 25659841

[79] Sun T Diaz FJ. Ovulatory Signals Alter Granulosa Cell Behavior Through YAP1 Signaling. Reprod Biol Endocrinol (2019) 1:113. 10.1186/s12958-019-0552-1 PMC6935177 31883523

[80] Fu D Lv X Hua G He C Dong J Lele SM. YAP Regulates Cell Proliferation, Migration, and Steroidogenesis in Adult Granulosa Cell Tumors. Endocr Relat Cancer (2014) 2:297–310. 10.1530/ERC-13-0339 PMC4222524 24389730

[81] Ye H Li X Zheng T Hu C Pan Z Huang J. The Hippo Signaling Pathway Regulates Ovarian Function *via* the Proliferation of Ovarian Germline Stem Cells. Cell Physiol Biochem (2017) 3:1051–62. 10.1159/000464113 28245464

[82] Tumaneng K Schlegelmilch K Russell RC Yimlamai D Basnet H Mahadevan N. YAP Mediates Crosstalk Between the Hippo and PI(3)K–TOR Pathways by Suppressing PTEN *via* miR-29. Nat Cell Biol (2012) 14(12):1322–9. 10.1038/ncb2615 PMC4019071 23143395

[83] Collak FK Yagiz K Luthringer DJ Erkaya B Cinar B. Threonine-120 Phosphorylation Regulated by Phosphoinositide-3-Kinase/Akt and Mammalian Target of Rapamycin Pathway Signaling Limits the Antitumor Activity of Mammalian Sterile 20-Like Kinase 1. J Biol Chem (2012) 28:23698–709. 10.1074/jbc.M112.358713 PMC3390644 22619175

[84] Kawamura K Cheng Y Suzuki N Deguchi M Sato Y Takae S. Hippo Signaling Disruption and Akt Stimulation of Ovarian Follicles for Infertility Treatment. Proc Natl Acad Sci USA (2013) 43:17474–9. 10.1073/pnas.1312830110 PMC3808580 24082083

[85] Suzuki N Yoshioka N Takae S Sugishita Y Tamura M Hashimoto S. Successful Fertility Preservation Following Ovarian Tissue Vitrification in Patients With Primary Ovarian Insufficiency. Hum Reprod (2015) 3:608–15. 10.1093/humrep/deu353 25567618

[86] Seo J Kim J. Regulation of Hippo Signaling by Actin Remodeling. BMB Rep (2018) 3:151–6. 10.5483/bmbrep.2018.51.3.012 PMC5882222 29353600

[87] Wen X Xie J Zhou L Fan Y Yu B Chen Q. The Role of Combining Medroxyprogesterone 17-Acetate With Human Menopausal Gonadotropin in Mouse Ovarian Follicular Development. Sci Rep (2018) 8:4439. 10.1038/s41598-018-22797-6 29535409 PMC5849710

[88] Edwards BS Isom WJ Navratil AM. Gonadotropin Releasing Hormone Activation of the mTORC2/Rictor Complex Regulates Actin Remodeling and ERK Activity in LβT2 Cells. Mol Cell Endocrinol (2016) 439:346–53. 10.1016/j.mce.2016.09.021 PMC5123956 27663077

[89] Jacinto E Loewith R Schmidt A Lin S Rüegg MA Hall A. Mammalian TOR Complex 2 Controls the Actin Cytoskeleton and Is Rapamycin Insensitive. Nat Cell Biol (2004) 11:1122–8. 10.1038/ncb1183 15467718

[90] Yi K Li R. Actin Cytoskeleton in Cell Polarity and Asymmetric Division During Mouse Oocyte Maturation. Cytoskeleton (Hoboken) (2012) 10:727–37. 10.1002/cm.21048 22753278

[91] Pennarossa G Gandolfi F Brevini T. Biomechanical Signaling in Oocytes and Parthenogenetic Cells. Front Cell Dev Biol (2021) 9:646945. 10.3389/fcell.2021.646945 33644079 PMC7905081

[92] Yu C Ji SY Dang YJ Sha QQ Yuan YF Zhou JJ. Oocyte-Expressed Yes-Associated Protein is a Key Activator of the Early Zygotic Genome in Mouse. Cell Res (2016) 3:275–87. 10.1038/cr.2016.20 PMC4783469 26902285

[93] Susor A Jansova D Cerna R Danylevska A Anger M Toralova T. Temporal and Spatial Regulation of Translation in the Mammalian Oocyte via the mTOR–eIF4F pathway. Nat Commun (2015) 6:6078. 10.1038/ncomms7078 25629602 PMC4317492

[94] Goto M Iwase A Ando H Kurotsuchi S Harata T Kikkawa F. PTEN and Akt Expression During Growth of Human Ovarian Follicles. J Assist Reprod Genet (2007) 11:541–6. 10.1007/s10815-007-9156-3 PMC3455024 17999178

[95] Lee SE Sun SC Choi HY Uhm SJ Kim NH. mTOR is Required for Asymmetric Division Through Small GTPases in Mouse Oocytes. Mol Reprod Dev (2012) 79:356–66. 10.1002/mrd.22035 22407942

[96] Lv X He C Huang C Wang H Hua G Wang Z. Timely Expression and Activation of YAP1 in Granulosa Cells is Essential for Ovarian Follicle Development. FASEB J (2019) 33:10049–64. 10.1096/fj.201900179RR PMC6704445 31199671

[97] Kovalevsky G Patrizio P. High Rates of Embryo Wastage With Use of Assisted Reproductive Technology: A Look at the Trends Between 1995 and 2001 in the United States. Fertil Steril (2005) 84(2):325–30. 10.1016/j.fertnstert.2005.04.020 16084872

[98] Patrizio P Sakkas D. From Oocyte to Baby: A Clinical Evaluation of the Biological Efficiency of in vitro fertilization. Fertil Steril (2009) 91(4):1061–6. 10.1016/j.fertnstert.2008.01.003 18325517

[99] Gleicher N Kushnir VA Barad DH. Worldwide Decline of IVF Birth Rates and its Probable Causes. Hum Reprod Open (2019) 2019(3):hoz017. 10.1093/hropen/hoz017 31406934 PMC6686986

[100] Ferraretti AP La Marca A Fauser BC Tarlatzis B Nargund G Gianaroli L. ESHRE Working Group on Poor Ovarian Response Definition. ESHRE Consensus on the Definition of ‘Poor Response’ to Ovarian Stimulation for in vitro fertilization: the Bologna criteria. Hum Reprod (2011) 26(7):1616–24. 10.1093/humrep/der092 21505041

[101] Gleicher N Kushnir VA Albertini DF Barad DH. Improvements in IVF in Women of Advanced Age. J Endocrinol (2016) 230(1):F1–6. 10.1530/JOE-16-0105 27154334

[102] Nelson SM Telfer EE Anderson RA. The Ageing Ovary and Uterus: New Biological Insights. Hum Reprod Update (2013) 19(1):67–83. 10.1093/humupd/dms043 23103636 PMC3508627

[103] Fauser BCJM. Patient-Tailored Ovarian Stimulation for *In Vitro* Fertilization. Fertil Steril (2017) 108(4):585–91. 10.1016/j.fertnstert.2017.08.016 28965553

[104] Mol BW Bossuyt PM Sunkara SK Garcia Velasco JA Venetis C Sakkas D. Personalized Ovarian Stimulation for Assisted Reproductive Technology: Study Design Considerations to Move From Hype to Added Value for Patients. Fertil Steril (2018) 109(6):968–79. 10.1016/j.fertnstert.2018.04.037 29935655

[105] Gatta V Tatone C Ciriminna R Vento M Franchi S d’Aurora M. Gene Expression Profiles of Cumulus Cells Obtained From Women Treated With Recombinant Human Luteinizing Hormone + Recombinant Human Follicle-Stimulating Hormone or Highly Purified Human Menopausal Gonadotropin Versus Recombinant Human Follicle-Stimulating Hormone Alone. Fertil Steril (2013) 99(7):2000–8.e1. 10.1016/j.fertnstert.2013.01.150 23472943

[106] Borgbo T Povlsen BB Andersen CY Borup R Humaidan P Grøndahl ML. Comparison of Gene Expression Profiles in Granulosa and Cumulus Cells After Ovulation Induction With Either Human Chorionic Gonadotropin or a Gonadotropin-Releasing Hormone Agonist Trigger. Fertil Steril (2013) 100(4):994–1001. 10.1016/j.fertnstert.2013.05.038 23856575

[107] Artini PG Tatone C Sperduti S D’Aurora M Franchi S Di Emidio G. Italian Society of Embryology, Reproduction and Research (SIERR). Cumulus Cells Surrounding Oocytes With High Developmental Competence Exhibit Down-Regulation of Phosphoinositol 1,3 Kinase/Protein Kinase B (PI3K/AKT) Signalling Genes Involved in Proliferation and Survival. Hum Reprod (2017) 32(12):2474–84. 10.1093/humrep/dex320 PMC5850344 29087515

[108] Cheng J Huang J Yuan S Zhou S Yan W Shen W. Circular RNA Expression Profiling of Human Granulosa Cells During Maternal Aging Reveals Novel Transcripts Associated With Assisted Reproductive Technology Outcomes. PloS One (2017) 12(6):e0177888. 10.1371/journal.pone.0177888 28644873 PMC5482436

